# Dietary risk factors for non-communicable diseases among Omani adults by latent class analysis and structural equation modelling

**DOI:** 10.1186/s40795-024-00987-y

**Published:** 2025-04-24

**Authors:** Adhra Al-Mawali, Ayaman Al-Harrasi, Avinash Daniel Pinto, Magdi Morsi, Abbas Balouchi, Francesco P. Cappuccio

**Affiliations:** 1 Centre of Studies & Research, Ministry of Health, Muscat, Sultanate of Oman; 2 Iran University of Medical Science, Tehran, Iran; 3 University of Warwick, WHO Collaborating Centre for Nutrition, Coventry, UK; 4 German University of Technology in Oman (GUtech), PO Box 1816, Athaibah, PC 130 Sultanate of Oman

**Keywords:** Dietary risk factors, Latent class analysis, Structural equation modelling, Oman, Non-communicable disease

## Abstract

**Background:**

Risk factor surveillance is vital for public health interventions in non-communicable diseases (NCD) control due to a noticeable nutrition transition among the population affecting dietary patterns. The objective was to investigate the dietary risk factors and its associations based on a first-of-its-kind analysis employing both Latent Class Analysis (LCA) and Structural equation modelling (SEM) to explore the hidden heterogeneity and subgroups with shared dietary pattern and to demonstrate the complex interaction of dietary factors with other risk factors in the development of NCDs.

**Methods:**

A cross-sectional survey was used. Secondary analysis of the 2017 Oman NCD Risk Factors Survey data was performed to investigate three major dietary risk factors (fruits and vegetables intake, eating out, and the type of oil used in cooking) of Omanis using LCA and SEM.

**Results:**

Dietary risk factors are prevalent in Omanis with 55.8% reporting intake of less than five fruit and vegetable servings per day, 45.3% ate outside the home 1–3 times per week, and 87.3% used vegetable oil for cooking. LCA showed two distinct classes of Omani population with majority belonging to the class mainly eating out 1–3 times per week, eating less than the recommended servings of fruits and vegetables, vegetable oil users, educated, and married young adults. SEM showed the intricate interplay of dietary factors with 8 direct paths and several indirect paths with NCD indicators.

**Conclusions:**

These findings may have important implications for targeting health promotion strategies among the high-risk group of Omanis identified in this analysis and inform decision makers for the reduction of NCDs.

**Supplementary Information:**

The online version contains supplementary material available at 10.1186/s40795-024-00987-y.

## Introduction

Globally, there is a nutrition transition characterized by a rise in the consumption of dense, processed, and convenience foods with a reduction in the intake of healthy food including fibre rich food. Dietary patterns and nutrient intake are affected by this nutrition transition, which in turn affects the risk of developing non-communicable diseases (NCDs) [1]. Based on global burden of diseases (GBD) reports, in 2019 dietary risk factors were attributed with 7.9 million deaths which is the cause of 30% of all NCDs-related deaths and 187.7 million DALYs globally [2].

The reduction of NCDs burden can be set in motion by targeting their predisposing risk factors including metabolic, behavioural, and environmental risk factors and by encouraging the increase of favourable behaviours, including consuming more fruit and vegetables and less salt. The reduction in NCDs is now a United Nations global health priority, advocated by the World Health Organization (WHO) Action Plan, targeting main risk factors like unhealthy diet, harmful alcohol consumption, tobacco use, and physical inactivity. A selection of cost-effective policy options (‘best buys’) has been identified of which promoting healthy consumption of food is one of them [3], since there is substantial evidence of a causal association between dietary habits and patterns, nutrient consumption and NCDs [4].

Healthy food includes a high intake of fibre rich food, not less than 5 servings of vegetables and fruits, low intake of saturated fat replaced by unsaturated fat, low salt intake, and low consumption of energy dense and processed food [5]. In addition to minerals, vitamins and antioxidants, fresh fruits and vegetables are considered to be rich sources of dietary fibre [6]. There is substantial empirical support indicating that the consumption of fruits and vegetables in quantities exceeding five servings (or 600 g) per day is efficacious in the prevention of various chronic diseases, including obesity, hypertension, cardiovascular diseases (CVD), and certain forms of cancer [7]. The prevalence of these diseases may be effectively gauged by monitoring trends in the consumption of these health-promoting foods.

Studies have shown that fruits and vegetables are not adequately consumed by most of the population residing in the Arab Gulf countries [5, 8]. In addition, it is known that some dishes traditionally consumed within the Arab Gulf region have a substantially high fat content (5% -20%) [5]. Fat is essential for energy, development, and vitamin absorption. However, it is advisable to maintain a moderate intake of fat. Based on its association to heart disease and its consequent impact on elevated blood cholesterol levels, dietary fats can be categorised into two types. Fat from animal sources, which can potentially raise blood cholesterol levels, is generally found in meat, whole milk, butter, cheese, chicken skin, and liver. Conversely, it is posited that fats derived from vegetable sources do not elevate blood cholesterol levels to the same extent as animal fats. However, the use of coconut oil and palm oil presents ambiguous evidence regarding their impact on cardiovascular health, in contrast to oils such as olive, sunflower, and canola. Despite this, fats are high in caloric content, and excessive consumption of fat-rich foods, such as fast food, may significantly contribute to the rising prevalence of obesity in the region. Consequently, it is advisable to satisfy energy requirements with plant-origin foods, including legumes, grains, seeds, and nuts. Although the Ministry of Health in Oman has endorsed the global objective to halt and reduce the impact of NCDs through the Oman National Strategy for the Prevention and Control of NCDs, which oversees the execution of targeted measures to lessen their burden [9], the FAO and the WHO continue to stress the importance of food-based dietary guidelines (FBDG).Hence, FBDG for the Arab Gulf countries was set up by the Arab Center for Nutrition for timely prevention and appropriate control of diet-related diseases, with a specific focus on chronic disease [5]. A recent modelling study suggests that the environmental sustainability of these national and global FBDG could be enhanced by reducing red and processed meat with balanced energy intake whilst increasing consumption of whole grains, fruits and vegetables, legumes, and nuts and seeds [10]. Realistic objectives for national programmes need to be set along with increased preventive measures and behavioural modification for adolescents and youth. This requires a multi-pronged approach that addresses their physical, social, and economic ecosystem. However, significant research gaps hinder our ability to design the most effective interventions and offset substantial economic costs. This is coupled with the lack of a robust method to measure the burden in order to inform these interventions poses a significant limitation, which is vital to produce lasting positive changes in dietary patterns and overall health outcomes.

In this study, we perform a secondary in-depth analysis on the data available from the STEPwise approach to risk factor surveillance (STEPS) survey, the largest representative survey on a national scale to identify the magnitude of NCD risk factors among the Omani population. In order to enable countries to gather core information on the main risk factors that contribute to disease burden, the WHO conceived the STEPS survey, with a flexible structure allowing countries to adapt it according to their specific needs [11]. It consists of three main steps: a questionnaire to assess socio-demographic, nutritional, and behavioural information; physical measurements; and biochemical measurements of blood glucose and lipid profile, including cholesterol. Nested within are further questions to identify levels of dietary intake, in particular on intake of fruits, vegetables and salt along with the consumption of food outside home and oils used in cooking.

The main objective of this study was to investigate the dietary risk factors of the Omani population using Latent Class Analysis (LCA) to explore the hidden heterogeneity and subgroups with shared dietary pattern to enlighten and inform decision makers with more reliable information that can guide them to build targeted action plans to reduce the burden of NCDs.

## Methods

### Sampling

Data was derived from the dataset of a large cross-sectional nationally-representative community-based Oman NCD Risk Factors Survey adopting WHO STEPwise methodology, and employing a multi-level stratified, geographically clustered sampling approach across all governorates (regions) of the Sultanate of Oman. Adjustments were done to the sample weights by primary and secondary sampling units as well as for household non-response level. Further details of the survey methodology are available on the main NCD survey article [12]. The analysis presented here included all eligible adult Omani citizens, men and women, aged 18 years and above taken from the total survey population (4,320 participants).

### Questionnaire

Based on the WHO STEPS instrument, a questionnaire for obtaining demographic and behavioural data in addition to the physical and biochemical measurements were used. A section on dietary history related to salt, fat, and fruit and vegetable intake was included in the questionnaire. The dietary questionnaire (Template in Text S1) was based on stated consumption. Respondents were provided with examples of foods high in salt content.

### Variables

The variables were described by two categories of variables – the dietary factors (dependent variables) and participant characteristics (independent variables). These variables which were categorised belong to biophysical indicators (waist-to-hip ratio, systolic blood pressure) and biochemical indicators (blood glucose, total cholesterol, high-density lipoprotein (HDL)) which were the variables collected as part of a big WHO NCD STEPwise survey conducted in Oman. Dietary intake was assessed through self-reported low consumption of fruits and vegetables by participants (representing intake of fruits and vegetables, which are critical components of a healthy diet), eating outside (used as a proxy for fast food consumption, which is often associated with unhealthy dietary choices due to less control over ingredients and cooking methods) and the type of oil used for cooking (saturated or unsaturated fat which can significantly affect health). Inadequate intake of fruits and vegetables was defined as intake of less than five portions (or 400g) of fruits and vegetables per day, as recommended minimal intake by the WHO [11]. In the course of the survey, the questionnaire included frequency in terms of days and servings to assess the consumption of fruits and vegetables. All the outcome variables were evaluated with the several independent variables employed in this study, including age, sex, education level, marital status, and work status.

### Statistical analysis

Statistical analysis was carried out with STATA (version 2016). Complex samples analysis was used to generate estimates with adjustment for the complex, multi-level sampling design, incorporating stratified sampling by governorates and enumerator areas. Descriptive analysis was calculated using proportions and testing relationships between categorical variables using chi square variance analysis at 95% confidence level. regression analysis then was performed to assess the significance of dietary behaviours on some unhealthy bio-physical and biochemical variables controlling for sociodemographic variables.

An analysis through Structural Equation Modelling (SEM) was used to assess the hypothesized relationships between the observed variables (dietary habits) and the latent variables (biophysical and biochemical indicators). SEM allows for the evaluation of both direct and indirect pathways, making it particularly useful for understanding complex relationships between diet and health outcomes. The standardized coefficients are used in the model to control measurement differences, providing a way to compare the strength and direction of associations across different variables uniformly. Model outcomes were evaluated through goodness of fit to ensure adequate data representation, along with showing how each dietary risk factor influences the biophysical and biochemical indicators both directly and indirectly.

In addition, there is a strong emphasis by many researchers on the signs and statistical significance of effects, but very little emphasis is often placed on the substantive and practical significance of the results. Using predicted or expected values to model hypothetical or prototypical cases can often result in more tangible results [13]. Thus, marginal effect analysis was conducted to extract more meaningful results. Moreover, although conventional variable-level studies (such as regression) include important information, important aspects of relationships that are often rooted in sample heterogeneity are not captured. Thus, LCA [14] was done which is one of several person-centred techniques that can be used to capture sample heterogeneity within and between various groups. By using STATA, LCA was performed on a weighted sample. The fact that a model is estimated and a case has been selected by random selection to fit into one specific class provides some information as to what response pattern the case will have – to understand how dietary patterns cluster among different demographic segments. The selection of the best model was guided by model fit indices such as Akaike’s Information Criterion (AIC) and Bayesian Information Criterion (BIC), with lower values indicating a better model fit. Maximum likelihood estimation using the expectation–maximization procedure, a well-known indicator of a statistical model's goodness of fit, is typically used to calculate the parameters of the subdistributions [14].

## Results

### Participant characteristics

Table 1 presents the demographic attributes of the study participants. 4320 Omani citizens were included in the secondary data analysis, of which the majority of participants were young in the age group of 18–44 years (59.39%), women (56.13%), had secondary education or above (68.44%), married (63.98%), and not currently working (62.72%).

**Table 1:** Socio-demographic attributes of the respondents, Oman STEPS survey, 2017

**Variable (n)**	**Proportion (%)**	**95% Confidence interval**
**Age group, years**		
18–29 (1,102)	33.7	31.3–36.2
30–44 (1,337)	25.7	23.8–27.8
40–49 (856)	19.0	17.0–21.1
50–59 (484)	10.7	9.2–12.5
60 + (542)	10.9	9.5–12.5
**Sex**		
Men (1,665)	43.9	41.4–46.4
Women (2,655)	56.1	53.6–58.6
**Education attainment**		
None (1,274)	21.8	20.1–23.7
Preparatory or less (487)	9.7	8.5–11.1
Secondary (1,648)	42.7	40.2–45.3
University or more (908)	25.7	23.6–28.0
**Marital status**		
Not married (742)	28.2	25.8–30.8
Married (3,161)	64.0	61.4–66.5
Separated/Divorced (114)	2.7	1.9–3.8
Widowed (303)	5.1	4.1–6.3
**Work status**		
Public sector (1,042)	23.9	21.9–26.0
Private sector (465)	13.4	11.6–15.5
Not working (2,810)	62.7	60.2–65.1

### Prevalence of dietary risk factors

More than half of the surveyed respondents reported taking less than five fruits and vegetable servings per day (Table 2) while 45.3% ate meals outside the home 1–3 times per week. It was also found that 87.3% used vegetable oil for cooking in comparison to butter (4.3%, 95% CI: 3.4%-5.6%) (Table 2). The prevalence of high dietary salt consumption was observed in 60.8% of the participants (Table 2).

**Table 2:** Prevalence of dietary risk factors

**Dietary variable**	**Proportion (%)**	**95% Confidence interval**
**High dietary salt**	60.8	44.6–74.9
**Fruit and/or vegetable intake**		
≥ 5 servings/day	44.2	41.7–46.7
< 5 servings/day	55.8	53.3–58.3
**Eating meals outside the home**		
Never	38.6	36.2–40.9
1–3 times per week	45.3	42.7–47.9
4 or more times per week	16.2	14.2–18.3
**Type of cooking oil used**		
Vegetable oil	87.3	85.7–88.8
Butter	4.3	3.4–5.6
Other	8.4	7.2–9.6

### Dietary risk factor associations

Fruits and vegetables intake varied significantly within the different age groups, education level groups, and marital status groups (all *p* < 0.001) (Table 3). The majority of respondents who had an intake of less than 5 servings of fruits and vegetables were young (18–29 years old) (37.4%), completed secondary school (42.2%) and were married (57.9%)).

**Table 3:** Prevalence of Fruits and Vegetables and its correlation to biophysical and biochemical risk factors of NCDs

	Fruits and vegetables intake % (95% CI)				*P* value^a^
	Column %		Row %		
	≥ 5	< 5	≥ 5	< 5	
Age group					< 0.001
18–29	29 (25.5–32.6)	37.4 (34–40.7)	38.1 (33.7–42.6)	61.9 (57.4–66.3)	
30–39	25.3 (22.2–28.4)	26.1 (23.5–28.6)	43.5 (39.2–47.7)	56.5 (52.3–60.8)	
40–49	21.6 (18.2–24.9)	16.9 (14.5–19.3)	50.3 (44.4–56.2)	49.7 (43.8–55.6)	
50–59	12.8 (9.71–15.8)	9.07 (7.49–10.7)	52.7 (44.8–60.6)	47.3 (39.4–55.2)	
60 +	11.3 (8.79–13.9)	10.6 (8.79–12.4)	45.8 (38.4–53.2)	54.2 (46.8–61.6)	
Sex					0.399
Men	43.68 (39.72 -47.72)	44.02 (40.83–47.25)	44 (40–48.1)	56 (51.9–60)	
Women	56.32 (52.28 -60.28)	55.98 (52.75- 59.17)	44.4 (41.2–47.5)	55.6 (52.5–58.8)	
Education level					< 0.001
No formal education	20.4 (17.6–23.3)	23 (20.6–25.4)	41.4 (36.8–45.9)	58.6 (54.1–63.2)	
Preparatory or less	10.2 (8.07–12.4)	9.31 (7.64–11)	46.5 (39.4–53.6)	53.5 (46.4–60.6)	
Secondary completed	42.9 (38.9–46.9)	42.6 (39.3–45.8)	44.4 (40.2–48.5)	55.6 (51.5–59.8)	
University +	26.5 (23–29.9)	25.2 (22.2–28.1)	45.5 (40.4–50.6)	54.5 (49.4–59.6)	
Marital Status					< 0.001
Never married	22.2 (18.6–25.8)	33 (29.7–36.4)	34.8 (29.5–40.1)	65.2 (59.9–70.5)	
Currently married	71.7 (67.9–75.4)	57.9 (54.5–61.2)	49.5 (46.6–52.4)	50.5 (47.6–53.4)	
Divorced/Separated	1.63 (0.74–2.51)	3.56 (2.09–5.04)	26.6 (13–40.1)	73.4 (59.9–87)	
Widowed	4.48 (2.95–6)	5.53 (4.03–7.04)	39.1 (28.4–49.7)	60.9 (50.3–71.6)	
Work status					0.805
Working in public sector	24.5 (21.1–27.8)	23.4 (20.8–25.9)	45.3 (40.4–50.2)	54.7 (49.8–59.6)	
Working in private sector	12.9 (9.79–16)	13.8 (11.3–16.4)	42.5 (34.4–50.6)	57.5 (49.4–65.6)	
Not working	62.6 (58.7–66.5)	62.8 (59.6–65.9)	44.2 (41–47.3)	55.8 (52.7–59)	
Blood pressure					0.974
SBP < 140 and DBP < 90	67.3 (63.4–71.2)	69 (65.9–72)	43.6 (40.7–46.5)	56.4 (53.5–59.3)	
SBP ≥ 140 and/or DBP ≥ 90 OR currently on meds	32.7 (28.8–36.6)	31 (28–34.1)	45.5 (40.7–50.3)	54.5 (49.7–59.3)	
BMI					0.565
1) BMI < 30	65.6(61.8–69.4)	67.5 (64.5–70.4)	43.5 (40.4–46.6)	56.5 (53.4–59.6)	
2) Obese BMI ≥ 30	34.4(30.6–38.2)	32.5 (29.6–35.5)	45.6 (41.3–49.9)	54.4 (50.1–58.7)	
Waist to Hip Ratio					0.089
Normal	33.9 (30.1–37.7)	39.6 (36.3–43)	40.7 (36.4–45)	59.3 (55–63.6)	
Abnormal	66.1 (62.3–69.9)	60.4 (57–63.7)	46.8 (43.6–49.9)	53.2 (50.1–56.4)	
Triglycerides					0.015
Triglycerides < 1.7	78.3 (75.1–81.4)	80 (77.4–82.7)	43.7 (40.8–46.5)	56.3 (53.5–59.2)	
Triglycerides ≥ 1.7	21.7 (18.6–24.9)	20 (17.3–22.6)	46.3 (40.8–51.8)	53.7 (48.2–59.2)	
Total cholesterol					< 0.001
Total Cholesterol ≥ 5.3	28.8 (25.2–32.4)	29.9 (26.8–32.9)	43.3 (38.6–48.1)	56.7 (51.9–61.4)	
Total Cholesterol < 5.3	71.2 (67.6–74.8)	70.1 (67.1–73.2)	44.6 (41.6–47.5)	55.4 (52.5–58.4)	
HDL					0.235
HDL ≥ 1.53	19.5 (16.7–22.4)	22.6 (19.9–25.3)	40.6 (35.6–45.7)	59.4 (54.3–64.4)	
HDL < 1.53	80.5 (77.6–83.3)	77.4 (74.7–80.1)	45.2 (42.3–48.1)	54.8 (51.9–57.7)	
Blood glucose					0.533
Blood glucose < 6.1	72.3 (68.8–75.8)	73.7 (71–76.3)	43.7 (40.7–46.7)	56.3 (53.3–59.3)	
Blood glucose ≥ 6.1 and < 7.0	11.4 (9.52–13.3)	12.5 (10.6–14.4)	42 (36.4–47.7)	58 (52.3–63.6)	
Blood glucose ≥ 7.0 or on diabetes medications	16.3 (13–19.5)	13.8 (11.7–15.9)	48.3 (41.5–55.1)	51.7 (44.9–58.5)	
Smoking					0.218
Currently not smoking	94.5 (92.7–96.4)	93 (91.4–94.5)	44.6 (42–47.2)	55.4 (52.8–58)	
Currently smoking	5.46 (3.62–7.29)	7.02 (5.46–8.58)	38.1 (28.3–47.9)	61.9 (52.1–71.7)	
Sedentary lifestyle					0.019
< 2 h	22.6 (19.1–26.1)	22.4 (19.9–24.9)	44.4 (39–49.7)	55.6 (50.3–61)	
2–3 h	36.7 (32.9–40.6)	36.2 (33.1–39.4)	44.5 (40.3–48.7)	55.5 (51.3–59.7)	
> 3 h	40.7 (36.8–44.5)	41.4 (38.2–44.6)	43.7 (39.8–47.6)	56.3 (52.4–60.2)	

^a^Chi square test was performed

Similarly, eating outside the home was associated with age, sex, education level, marital status and working status (all p < 0.001). Most respondents eating more than 4 times/week were 18–29 years (56.4%), men (64.9%), and completed secondary school (48.8%), never married (30.1%), and not currently working (47.4%). Most of the women ate at home while most of the men eat 1–3 times/week outside the home. In general, as the education level increased, there was a higher tendency for eating outside the home. Most of those who were working were eating from outside but those who were not working ate mostly at home (Table 4).

**Table 4:** Prevalence of fast food and its correlation to biophysical and biochemical risk factors of NCDs

	Meal out % (95% CI)						*P* value^a^
	Column %			Row %			
	0 times	1 to 3 times	> 4 times	0 times	1 to 3 times	> 4 times	
Age group							< 0.001
18–29	23 (20.2–25.9)	35.4 (31.4–39.4)	56.4 (49.5–63.3)	26.1 (22.6–29.7)	47.1 (42.4–51.8)	26.8 (22.4–31.2)	
30–39	20.9 (18.6–23.2)	29.8 (26.3–33.2)	26.3 (20.7–32)	31.3 (27.8–34.7)	52.3 (48–56.5)	16.5 (13–19.9)	
40–49	21.7 (19–24.4)	20.6 (17–24.2)	8.74 (5.33–12.2)	43.8 (38.1–49.4)	48.9 (42.8–54.9)	7.38 (4.51–10.3)	
50–59	14.3 (11.7–16.8)	8.88 (6.48–11.3)	6.15 (1.56–10.7)	52.4 (43.9–60.8)	38.2 (29.9–46.4)	9.44 (2.6–16.3)	
60 +	20.1 (17.1–23.1)	5.35 (3.59–7.1)	2.4 (1.16–3.65)	73.4 (66.7–80.1)	22.9 (16.3–29.5)	3.67 (1.78–5.56)	
Sex							< 0.001
Men	33.5 (30.2–36.7)	46 (41.9–50.2)	64.9 (57.8–72.1)	29.2 (25.8–32.5)	47.1 (43.1–51.2)	23.7 (20.3–27.1)	
Women	66.5 (63.3–69.8)	54 (49.8–58.1)	35.1( 27.9–42.2)	46 (42.9–49.2)	43.8 (40.5–47.1)	10.2 (7.63–12.7)	
Education level							< 0.001
No formal education	38.8 (35.6–42.1)	12.5 (10.2–14.8)	5 (2.81–7.19)	69.9 (65.6–74.2)	26.4 (22.2–30.5)	3.77 (2.15–5.39)	
Preparatory or less	11.8 (9.82–13.7)	9.18 (6.92–11.4)	5.58 (3.07–8.08)	47.3 (40.2–54.5)	43.3 (35.8–50.7)	9.38 (5.32–13.4)	
Secondary completed	33.3 (30.2–36.4)	49.1 (45–53.2)	48.8 (41.7–55.9)	29.9 (26.6–33.2)	51.8 (47.6–55.9)	18.3 (14.9–21.8)	
University +	16.1 (13.4–18.8)	29.3 (25.6–32.9)	40.6 (33.7–47.6)	23.9 (19.9–27.9)	50.9 (45.8–56)	25.2 (20.5–29.9)	
Marital Status							< 0.001
Never married	17.3 (14.3–20.3)	29.2 (25.3–33.1)	53 (46.1–60)	23.5 (19.2–27.7)	46.5 (41–52)	30.1 (24.8–35.4)	
Currently married	71.5 (68.2–74.9)	64.9 (60.9–69)	44.6 (37.7–51.4)	43 (40.2–45.7)	45.8 (42.9–48.7)	11.2 (9.3–13.1)	
Divorced/Separated	2.25 (1.32–3.17)	3.21 (1.42–5.01)	1.74 (0.453–3.02)	33.3 (18.9–47.6)	55.9 (39.3–72.6)	10.8 (2.75–18.8)	
Widowed	8.89 (6.89–10.9)	2.6 (1.24–3.95)	0.626 (0.053–1.2)	72.9 (62.1–83.7)	25 (14.2–35.8)	2.15 (0.179–4.11)	
Work status							< 0.001
Working in public sector	15.8 (13.5–18.1)	27.6 (24.2–31)	34.2 (27.8–40.7)	25.3 (21.5–29)	51.8 (47–56.7)	22.9 (18.6–27.3)	
Working in private sector	10.2 (7.92–12.4)	14.9 (11.4–18.3)	18.4 (13–23.7)	28.8 (22.4–35.3)	49.4 (41.3–57.5)	21.8 (15.4–28.1)	
Not working	74 (71.1–77)	57.5 (53.5–61.6)	47.4 (40.3–54.5)	45.9 (42.8–48.9)	41.8 (38.6–45)	12.3 (9.84–14.8)	
Blood pressure							0.003
SBP < 140 and DBP < 90	63.3 (59.9–66.7)	70.1 (66.1–74.1)	76.3 (70.7–81.9)	35.7 (33.1–38.2)	46.4 (43.4–49.3)	18 (15.4–20.6)	
SBP ≥ 140 and/or DBP ≥ 90 OR currently on meds	36.7 (33.3–40.1)	29.9 (25.9–33.9)	23.7 (18.1–29.3)	44.9 (40.3–49.6)	42.9 (38–47.9)	12.1 (9.16–15.1)	
BMI							0.003
1) BMI < 30	61.8 (58.5–65.2)	67.9 (64.2–71.6)	67.9 (64.2–71.6)	35.7 (33–38.4)	46 (42.9–49.2)	18.2 (15.6–20.9)	
2) Obese BMI ≥ 30	38.2 (34.8–41.5)	32.1 (28.4–35.8)	32.1 (28.4–35.8)	44.3 (40.1–48.5)	43.8 (39.4–48.1)	11.9 (8.74–15.1)	
Waist to Hip Ratio							0.001
Normal	30.9 (27.8–34)	40.3 (36.2–44.5)	45.6 (38.4–52.8)	31.6 (28–35.1)	48.3 (43.8–52.7)	20.2 (16.2–24.1)	
Abnormal	69.1 (66–72.2)	59.7 (55.5–63.8)	54.4 (47.2–61.6)	42.5 (39.5–45.6)	43 (39.8–46.2)	14.5 (12.1–16.9)	
Triglycerides							0.041
Triglycerides < 1.7	76.3 (73.2–79.3)	82.5 (79.5–85.5)	77.5 (71.5–83.5)	37.1 (34.6–39.6)	47.1 (44.2–50)	15.8 (13.5–18.1)	
Triglycerides ≥ 1.7	23.7 (20.7–26.8)	17.5 (14.5–20.5)	22.5 (16.5–28.5)	44.2 (38.8–49.6)	38.2 (32.8–43.7)	17.6 (12.9–22.3)	
Total cholesterol							< 0.001
Total cholesterol ≥ 5.3	36.8 (33.6–40.1)	25.3 (21.7–29)	22.1 (15.6–28.6)	48.6 (43.8–53.3)	39.2 (34.4–44)	12.2 (8.36–16)	
Total cholesterol < 5.3	63.2 (59.9–66.4)	74.7 (71–78.3)	77.9 (71.4–84.4)	34.4 (31.9–37)	47.8 (44.8–50.8)	17.8 (15.3–20.2)	
HDL							< 0.001
HDL ≥ 1.53	24.7 (21.9–27.5)	21.2 (17.9–24.4)	12.4 (8.31–16.6)	45.1 (40.1–50.1)	45.4 (40.1–50.7)	9.51 (6.38–12.6)	
HDL < 1.53	75.3 (72.5–78.1)	78.8 (75.6–82.1)	87.6 (83.4–91.7)	36.8 (34.2–39.4)	45.2 (42.3–48.2)	17.9 (15.5–20.4)	
Blood glucose							< 0.001
Blood glucose < 6.1	66.6 (63.5–69.7)	75.2 (71.7–78.8)	83.5 (79.3–87.8)	35.1 (32.4–37.7)	46.5 (43.5–49.6)	18.4 (15.8–21)	
Blood glucose ≥ 6.1 AND < 7.0	13.3 (11.4–15.1)	11.9 (9.63–14.2)	10.1 (6.55–13.6)	42.2 (36.7–47.7)	44.4 (38.5–50.4)	13.4 (9–17.7)	
Blood glucose ≥ 7.0 or on diabetes medications	20.1 (17.3–23)	12.8 (9.78–15.9)	6.43 (4.01–8.84)	53.1 (46.2–60.1)	39.8 (32.6–46.9)	7.09 (4.46–9.73)	
Smoking							< 0.001
Currently not smoking	96.1 (94.6–97.6)	93.8 (91.9–95.6)	87.3 (83.2–91.4)	39.6 (37.2–42)	45.4 (42.7–48)	15.1 (13–17.2)	
Currently smoking	3.91 (2.45–5.38)	6.22 (4.38–8.05)	12.7 (8.55–16.8)	23.7 (15.6–31.8)	44.1 (34.4–53.9)	32.2 (23.1–41.2)	
Sedentary lifestyle							< 0.001
< 2 h	22.6 (19.6–25.6)	24.6 (21.1–28.1)	13.8 (9.52–18)	39.4 (34.4–44.5)	50.5 (45.2–55.9)	10 (6.97–13.1)	
2–3 h	34.3 (31.2–37.4)	37.7 (33.8–41.7)	38.6 (31.5–45.8)	36.2 (32.5–39.9)	46.8 (42.5–51)	17 (13.3–20.7)	
> 3 h	43.1 (39.8–46.4)	37.7 (33.6–41.7)	47.6 (40.5–54.7)	40.2 (36.6–43.8)	41.3 (37.2–45.3)	18.5 (15.3–21.8)	

^a^Chi square test was performed

We found that the type of oil used for cooking varied significantly among sex, educational level, and working status groups (all p < 0.001). Most respondents who used vegetable oils were mostly women (56.2%), completed secondary school (42.3%) and not working (61.9%). Among all subcategories of the above which showed significant correlation with the type of cooking oil used, vegetable oil was the main oil used for cooking (Table 5).

**Table 5:** Prevalence of type of oil used of cooking and its correlation to biophysical and biochemical risk factors of NCDs

	Type of cooking oil % (95% CI)						*P* value^a^
	Column %			Row %			
	Veg	Butter	Others	Veg	Butter	Others	
Age group							0.084
18–29	33.6 (30.9–36.2)	40.6 (26.9–54.3)	31.6 (24.9–38.3)	86.9 (83.9–90)	5.23 (2.73–7.72)	7.82 (5.86–9.79)	
30–39	25.9 (23.8–28)	16.7 (9.52–24)	28.2 (20.7–35.7)	88 (84.9–91.1)	2.83 (1.64–4.01)	9.18 (6.27–12.1)	
40–49	19.1 (16.9–21.3)	24 (13.1–34.9)	15.1 (10.9–19.4)	87.9 (84.6–91.2)	5.47 (2.72–8.22)	6.65 (4.7–8.61)	
50–59	10.8 (8.99–12.6)	9.31 (2.64–16)	10.6 (6.6–14.6)	88 (83.8–92.2)	3.77 (1.02–6.51)	8.27 (5.02–11.5)	
60 +	10.6 (8.96–12.3)	9.33 (4.39–14.3)	14.5 (9.87–19.1)	85.2 (81–89.3)	3.71 (1.81–5.62)	11.1 (7.45–14.8)	
Sex							< 0.001
Men	43.8 (41.1–46.5)	38.9 (26.5–51.3)	46.2 (39–53.4)	87.3 (85.1–89.5)	3.85 (2.37–5.33)	8.82 (7.16–10.5)	
Women	56.2 (53.5–58.9)	61.1 (48.7–73.5)	53.8 (46.6–61)	87.3 (85.1–89.5)	4.71 (3.13–6.29)	7.99 (6.3–9.68)	
Education level							< 0.001
No formal education	21.6 (19.6–23.6)	20.6 (12.9–28.4)	24.9 (19.1–30.8)	86.3 (83.6–89.1)	4.1 (2.66–5.54)	9.56 (7.18–11.9)	
Preparatory or less	9.72 (8.3–11.1)	14.4 (4.95–23.8)	7.32 (4.3–10.3)	87.3 (82.4–92.2)	6.42 (2.05–10.8)	6.29 (3.67–8.91)	
Secondary completed	42.3 (39.6–45.1)	35.9 (22.7–49)	50.1 (42.7–57.4)	86.6 (84.1–89)	3.64 (1.91–5.38)	9.8 (7.98–11.6)	
University +	26.4 (24–28.8)	29.1 (17–41.2)	17.7 (10.1–25.2)	89.4 (85.9–92.9)	4.9 (2.51–7.28)	5.72 (2.97–8.47)	
Marital Status							0.162
Never married	27.9 (25.2–30.6)	36 (22.4–49.7)	27.9 (21.3–34.6)	86.2 (82.7–89.7)	5.53 (2.76–8.3)	8.25 (5.94–10.6)	
Currently married	64 (61.2–66.8)	58.5 (45.1–71.8)	66.6 (59.7–73.5)	87.3 (85.5–89.2)	3.96 (2.78–5.14)	8.7 (7.19–10.2)	
Divorced/Separated	2.76 (1.74–3.78)	1.77 (-0.277–3.81)	2.72 (0.449–4.98)	88.8 (81–96.7)	2.82 (0.87–8.80)	8.37 (1.3–15.4)	
Widowed	5.32 (4.1–6.53)	3.74 (0.66–6.83)	2.74 (1.06–4.42)	92.2 (88.3–96.1)	3.23 (0.593–5.86)	4.55 (1.71–7.38)	
Work status							< 0.001
Working in public sector	23.8 (21.6–26)	20.3 (9.67–30.9)	25.5 (20–30.9)	87.4 (84.5–90.2)	3.7( 1.57–5.82)	8.94 (6.95–10.9)	
Working in private sector	14.3 (12.1–16.5)	10.7 (2.54–18.9)	6.37 (3.29–9.45)	92.6 (89.2–96)	3.46 (0.699–6.21)	3.95 (1.98–5.93)	
Not working	61.9 (59.3–64.6)	69 (56.8–81.2)	68.1 (62.1–74.2)	86.2 (84–88.3)	4.77 (3.33–6.21)	9.06 (7.38–10.7)	
Blood pressure							0.048
SBP < 140 and DBP < 90	67.9 (65.2–70.5)	74.4 (63.8–85)	68.3 (62.2–74.5)	86.9 (84.9–88.9)	4.73 (3.3–6.16)	8.37 (6.83–9.92)	
SBP ≥ 140 and/or DBP ≥ 90 OR currently on meds or diagnosed by a physician	32.1 (29.5–34.8)	25.6 (15–36.2)	31.7 (25.5–37.8)	88.2 (85.8–90.5)	3.49 (1.91–5.07)	8.32 (6.55–10.1)	
BMI							0.019
1) BMI < 30	67.3 (64.7–69.8)	69.4 (57.9–80.8)	57.7 (50.6–64.8)	88.2 (86.3–90.2)	4.52 (3.09–5.94)	7.24 (5.79–8.69)	
2) Obese BMI ≥ 30	32.7 (30.2–35.3)	30.6 (19.2–42.1)	42.3 (35.2–49.4)	85.5 (82.8–88.1)	3.97 (2.3–5.64)	10.6 (8.47–12.7)	
Waist to Hip Ratio							< 0.001
Normal	38.1 (35.4–40.8)	40.1 (26.5–53.7)	23.7 (15.5–32)	90.1 (87.2–93.1)	4.68 (2.58–6.77)	5.18 (3.01–7.35)	
Abnormal	61.9 (59.2–64.6)	59.9 (46.3–73.5)	76.3 (68–84.5)	86.1 (84.2–88)	4.1 (2.79–5.42)	9.77 (8.3–11.2)	
Triglycerides							0.161
Triglycerides < 1.7	78.7 (76.5–81)	85.4 (76.1–94.8)	81.4 (76.2–86.6)	86.7 (84.9–88.6)	4.67 (3.4–5.95)	8.58 (7.19–9.97)	
Triglycerides ≥ 1.7	21.3 (19–23.5)	14.6 (5.2–23.9)	18.6 (13.4–23.8)	89.5 (86.5–92.4)	3.04 (0.957–5.13)	7.49 (5.28–9.7)	
Total cholesterol							0.066
Total Cholesterol ≥ 5.3	29.9 (27.4–32.4)	26.3 (15.6–36.9)	26.6 (19–34.2)	88.6 (85.6–91.6)	3.87 (2.15–5.58)	7.55 (4.97–10.1)	
Total Cholesterol < 5.3	70.1 (67.6–72.6)	73.7 (63.1–84.4)	73.4 (65.8–81)	86.8 (84.9–88.6)	4.53 (3.15–5.92)	8.69 (7.38–10)	
HDL							0.696
HDL ≥ 1.53	21.1 (19–23.2)	17.3 (9.49–25.1)	25.1 (17.5–32.7)	86.6 (82.9–90.3)	3.53 (1.93–5.13)	9.85 (6.41–13.3)	
HDL < 1.53	78.9 (76.8–81)	82.7 (74.9–90.5)	74.9 (67.3–82.5)	87.5 (85.8–89.2)	4.55 (3.22–5.88)	7.95 (6.76–9.14)	
Blood glucose							0.007
Blood glucose < 6.1	73 (70.6–75.3)	75.7 (66.6–84.8)	72.4 (66.5–78.3)	87.2 (85.3–89.2)	4.49 (3.09–5.9)	8.28 (6.82–9.74)	
Blood glucose ≥ 6.1 AND < 7.0	11.7 (10.2–13.2)	15.9 (9.06–22.8)	13.4 (9.13–17.7)	85 (81.2–88.7)	5.73 (3.39–8.07)	9.32 (6.32–12.3)	
Blood glucose ≥ 7.0 or on diabetes medications	15.3 (13.3–17.3)	8.37 (2.24–14.5)	14.1 (9.68–18.6)	89.6 (86.5–92.8)	2.44 (0.622–4.25)	7.93 (5.31–10.6)	
Smoking							< 0.001
Currently not smoking	94.4 (93.2–95.6)	90.7 (82.8–98.6)	88.7 (84.4–93)	87.9 (86.3–89.5)	4.19 (3.08–5.31)	7.91 (6.69–9.12)	
Currently smoking	5.61 (4.39–6.84)	9.3 (1.4–17.2)	11.3(6.96–15.6)	78.5 (70.9–86)	6.45 (0.911–12)	15.1 (9.23–21)	
Sedentary lifestyle							0.003
< 2 h	22.4 (20.1–24.6)	24.2 (14–34.4)	22.8 (16.8–28.7)	86.9 (83.8–90)	4.64 (2.55–6.74)	8.47 (6.05–10.9)	
2–3 h	35.9 (33.3–38.5)	33.3 (21.2–45.5)	44.5 (37.1–51.9)	85.9 (83–88.7)	3.94 (2.25–5.63)	10.2 (7.86–12.6)	
> 3 h	41.7 (39.1–44.4)	42.5 (29.2–55.8)	32.7 (26.2–39.3)	88.9 (86.5–91.2)	4.46 (2.55–6.37)	6.68 (5.16–8.2)	

^a^Chi square test was performed

Those having high blood pressure were mostly eating outside the home (55%). However, most of those who were eating more than 4 times outside had normal systolic blood pressure. They mostly used vegetable oil for cooking (88.2%). Fruits and vegetables did not vary significantly between blood pressure groups.

**Fig. 1:**
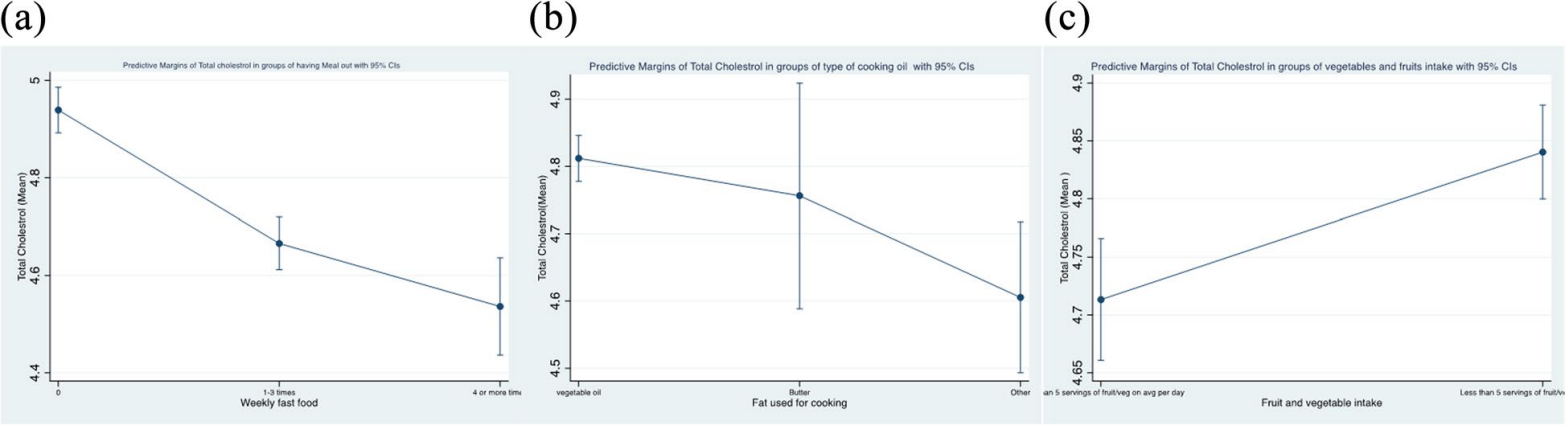
Predictive Margins of Total Cholesterol by group of main dietary risk factors (**a**) by having meals out; (**b**) by type of cooking oil; (**c**) by fruit and vegetable consumption

Most of those who are eating outside more than 4 times/week and those using vegetable oil for cooking are having normal weight but abnormal waist-to-hip ratio. Higher total cholesterol is seen more with those who are eating at home and eating less than five servings of fruits and vegetables but it is not correlated significantly with type of oil used for cooking. Interestingly, low HDL was seen mostly among those who ate more than 4 times/week outside compared to those who ate a home. We found that high blood glucose is found more in vegetable oil users and those who are eating at home. Regressing total cholesterol on the dietary risk factors and controlling for sociodemographic (age, sex, work status, marital status, education level, family history of hypercholesteremia), and biophysical factors (obesity, waist-to-hip ratio) we found that dietary risk factors are significant indicators for total cholesterol level (Table 6). Further analysis on margins, we found that higher total cholesterol level is associated with eating less fruits and vegetables, using vegetable oil for cooking, and eating at home (Fig. 1A, B, C).

**Table 6:** Output of regression model of Total cholesterol on dietary risk factors controlling for sociodemographic and biophysical factors

**Covariates**	**Coefficient (× 10**^**–3**^**)**	**95% Confidence intervals (× 10**^**–3**^**)**	***p*-value**
Age (years)	7.9	4.6–11.1	< 0.001
Sex (m&f)	130.6	48.4–212.8	0.002
Marital status^a^	35.7	-21.5–93.0	0.221
Educational levels^a^	-56.1	-97.6–14.6	0.008
Working status^a^	-131.2	-180.0–82.4	< 0.001
Smoking	105.7	-41.6–253.0	0.16
Eating meal out^b^	-223.9	-275.8–172.1	< 0.001
Oil used for cooking^b^	-100.8	-157.4–44.3	< 0.001
Family history of high cholesterol (y/n)	120.5	47.1–193.8	0.001
Fruit and vegetable intake^b^	125.8	59.1–192.5	< 0.001
Waist-to-hip ratio	89.9	18.4–161.5	0.014
Obesity	87.9	19.2–156.6	0.012
Sedentary lifestyle	12.7	-52.9–78.3	0.704

The coefficient value signifies how much the mean of the dependent variable (total cholesterol) changes given a one-unit shift in the independent variable (participant characteristics) while holding other variables in the model constant

^a^Units in Table 1

^b^Units in Table 2

### Latent class analysis

We compared two models for identifying possible population subgroups using LCA analysis and we selected the two-class model as it had a lower Akaike’s Information Criterion (AIC) and Bayesian Information Criterion (BIC) (Table S1). Using the probabilistic rules based on these two criteria in LCA, we found that there are two distinct subgroups of dietary habits (Table 7), where most Omanis (90%) are more likely to be in class 2. In other words, each Omani is having 90% probability of being in class 2. Table 8 presents Class 2 which are those who are eating less than the recommended servings of fruits and vegetables, following sedentary lifestyle, using vegetable oil for cooking, and mostly eating at home. Putting the dietary habits as indicators for waist-to-hip ratio in the LCA, we found that those in class 2 were with higher probability of having abnormal waist-to-hip ratio (64%) (Table S2, S3).

**Table 7:** Latent class marginal probabilities for dietary subgroups (× 10^–2^)

**Class**	**Margin**	**Standard error**	**95% Confidence intervals**
1	9.95	3.57	4.82–19.43
2	90.04	3.57	80.56–95.18

**Table 8:** Latent class marginal means for dietary habits (× 10^–2^)

	**Class 1**		**Class 2**	
	Margin	95% Confidence intervals	Margin	95% Confidence intervals
**Eating outside the home (times per week)**				
Never	0.00013		58.1	53.3–62.8
1–3 times	79.6	68.4–87.5	32.1	28.2–36.3
4 or more times	20.4	12.5–31.6	9.7	8.3–11.4
**Number of serving of fruit and/or vegetables per day**				
> 5	20.6	12.6–31.9	39.2	37.3–41.2
< 5	79.4	68.1–87.4	60.8	58.8–62.7
**Fat used for cooking**				
Vegetable oil	62.6	41.3–79.8	90.6	89.4–91.7
Butter	0.49	0–98.9	4.0	3.3–4.8
Other	37.0	18.6–60.1	5.4	4.5–6.4

When the LCA was extended to include sociodemographic factors with the dietary factors, we found 2 distinct classes with each Omani having a 67% probability of being in Class 1 which were those in the 18–39 age group, completed secondary school, married, not working, eating less than the recommended servings of fruits and vegetables, having sedentary lifestyle, eating outside for 1–3 times/week, and using vegetable oil for cooking (Tables S4, S5). Regressing blood glucose on the above classes’ variables using LCA, we found that on condition of categorised in Class 1, an individual would have a 21% and 6% probability of developing pre-diabetes and diabetes, respectively (Tables S6, S7). However, being in Class 2 (being married, above 50 years of age, not having formal education, not working, having sedentary lifestyle, eating outside the home, and eating less serving of fruits and vegetables, and using vegetable oil for cooking), an individual would have a 32% probability of developing diabetes. As a result, we can see that increasing age is a strong indicator for diabetes as it raises the probability of developing diabetes from 6 to 32% despite the individual having almost the same dietary habits. Thus, most Omani citizens in Class 1 would have a higher probability of developing diabetes if they keep the same lifestyle habits as they are aging.

### Direct effects of dietary risk factors

**Fig. 2:**
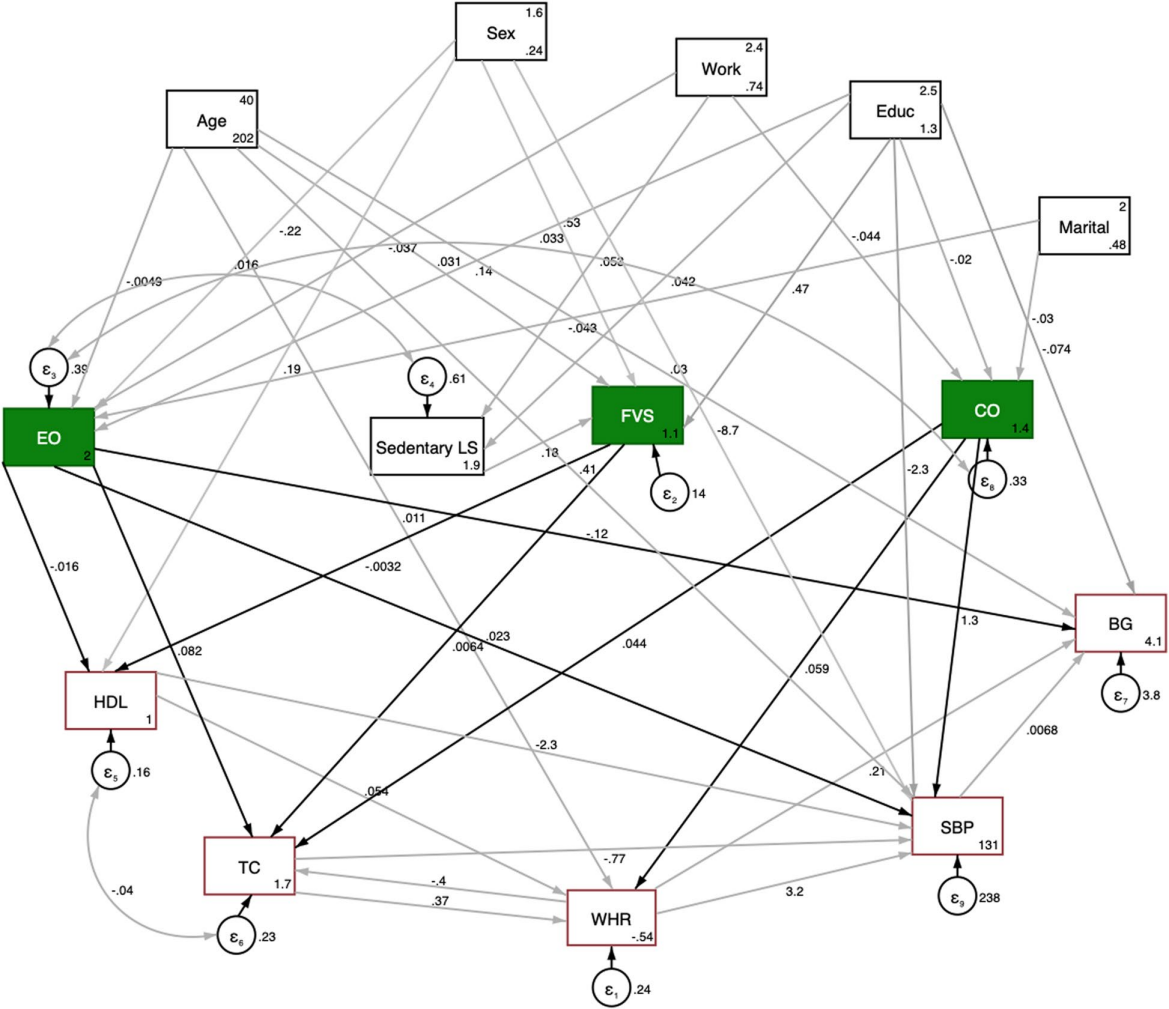
Path analysis diagram with standardized estimates illustrating structural Equation Modelling (SEM) of dietary risk factor associations. *box indicates observed variable; straight line with one arrowhead denotes direct effect. Green boxes denote variables with direct effects on dietary risk factors; ** EO: Eating Outside the home, LS: Lifestyle, FVS: Fruit & vegetable servings, CO: Cooking oil used; Educ: Education level; Marital: Marital status, HDL: High-density lipoprotein, TC: Total cholesterol, WHR: waist-to-hip ratio, SBP: systolic blood pressure, BG: blood glucose

Using Structural equation modelling, we proposed the model in Fig. 2 which showed the standardized coefficients to control for measurement differences. The model showed good fit (Table S8), which suggests that hypothesised model accurately represents the data. Table 9 shows how each dietary risk factor influences the biophysical and biochemical indicators both directly and indirectly. It was found that dietary risk factors have a direct effect through 8 paths on main biophysical (Waist-to-hip ratio, Systolic blood pressure) and main biochemical (blood glucose, total cholesterol, and HDL) risk indicators of NCDs along with several indirect effects (Table 9). Eating outside the home had a direct and indirect effect on all measured indicators, influencing systolic blood pressure and Waist-to-Hip ratio. There is a direct effect on total cholesterol and negatively influencing HDL. Fruit/Vegetable intake was generally associated with better outcomes such as lower Waist-to-Hip ratio and more favourable cholesterol levels. The fat used for cooking affected all parameters with both direct and indirect effects, showing significant influence on Waist-to-Hip ratio and systolic blood pressure.

**Table 9:** Direct and indirect effect of dietary risk factors on the main biophysical and biochemical parameters (× 10^–2^)

	**Waist-to-hip ratio**	**Total cholesterol**	**Blood glucose**	**High-density lipoprotein**	**Systolic blood pressure**
**Eating outside the home (times per week)**					
Total	3.73	10.61	-3.71	-2.63	0.33
Direct	12.70	12.15	-3.90	7.32	0.09
Indirect	3.73	-1.54	0.20	84.10	0.25
**Number of serving of fruit and/or vegetables per day**					
Total	1.51	4.53	0.08	-2.91	0.19
Direct	7.30	5.15	35.00	-2.91	7.10
Indirect	1.51	-0.62	0.08	9.20	0.19
**Fat used for cooking**					
Total	7.95	2.15	0.67	15.00	4.91
Direct	7.17	5.42	26.00	7.00	4.28
Indirect	0.78	-3.27	0.67	9.30	0.63

## Discussion

Relatively few studies have explored the spectrum of dietary risk behaviors among Omanis and their interplay with biophysical and biochemical risk factors in the etiology of NCDs. This investigation reveals that diet-related risk factors for NCDs are widespread in Oman, paralleling trends observed globally, and display variation across age, sex, and employment status. The intake of fruits and vegetables significantly varies both among and within nations, heavily influenced by economic, cultural, and agricultural contexts, yet remains suboptimal in many regions [15]. Notably, over half of the Omani population consumes fewer than the recommended five daily servings of fruits and vegetables, and a substantial portion frequently eats meals outside the home (1–3 times per week). Furthermore, this study identified that 60% of Omanis exceed the recommended minimum salt intake, corroborated by a 24-h urine analysis which also indicated that fewer than 10% of participants met the WHO potassium excretion targets [16].

Consumption of fruit and vegetables may mitigate the risk of NCDs through the enhanced availability of an assortment of nutrients and their ability to regulate associated risk factors. It has been shown that nutrients provided by fruits and vegetables lower blood pressure and cholesterol which are considered risk factors for cardiovascular disease and stroke [17, 18]. We found that a low intake of fruits and vegetables was strongly associated with raised cholesterol level in Omanis, however it showed no significant effect on blood pressure. The non-significance of the effect on blood pressure might be due to the lack of time series data which are more capable to pick this effect than cross-sectional data used in this study.

Dietary fibre can also help to control insulin levels which could have an effect on the risk of developing type 2 diabetes [19]. Our study observed that a low intake of fruit and vegetables was not directly associated with raised blood glucose, though by LCA we found that among other dietary risk factors and sociodemographic features (but not in isolation), it increases the probability of having high blood glucose. Increased fibre intake, in addition to high water content of fruit and vegetables, can help reduce the risk of obesity by supporting satiety and reducing hunger thereby limiting overall energy consumption [20]. Supporting this, we found that a low intake of fruits and vegetables was strongly associated with an abnormal waist-to-hip ratio (abdominal obesity) although with no significant correlation with general obesity.

Diets which include energy-dense, highly-refined foods and processed starches contribute to overweight and obesity, which in turn is associated with increased all-cause mortality and elevated risk of disease or death from cardiovascular disease, diabetes, and various types of cancer [21]. It does so by raising blood pressure, insulin resistance, and blood cholesterol as well as hormone levels [21]. Several determinants with regard to fruits and vegetable consumption in various populations worldwide have been indicated, including preferences, ethnicity, availability, affordability and cultural variations. Most of those who have low intake of fruits and vegetables in the population of Omanis are young and educated (at least completed secondary school). This might alarm health promotion programmes offered to this group which are often challenging to educate and often in need for modern, updated, and innovative channels for health education outreach. Availability and affordability might not be an issue in a country like Oman, but a comparably low affordability and high availability of energy dense food might reduce the intake of fruits and vegetables [22].

Consumption of fast food frequently is largely unhealthy and leads to weight gain, obesity, type 2 diabetes, and heart disease [23, 24]. Fast food typically has a high-energy density, which, coupled with bigger portions, prompts overconsumption of calories [25]. The Cardia study done on American population suggests that regular consumption of fast-food is positively correlated with increased weight gain and extended risk of insulin resistance over a 15 years’ duration. Individuals who consumed fast food for more than two times per week gained 4.5 kg in weight and had an insulin resistance increase of 104% when compared to individuals eating less than one fast food meal per week [26]. We assume that a majority of those eating outside the home are consuming fast food due to its higher availability and affordability. Our study revealed that most Omanis are eating 1–3 times/week outside the home, and are largely young and educated males. Further analysis discovered that among those who with prevailing high blood pressure and high waist-to-hip ratio, most of them ate outside the home. However, on the contrary, we found that raised total cholesterol and raised blood glucose was associated more with those who ate at home. This can be explained by the availability and affordability of ready-to-make high calorie, processed food easily prepared at home which is similar to fast food prepared at restaurants. Other studies have also found associations with unhealthy diets in men and younger people [27, 28]. This could possibly be owed to the migration among the youth to the main cities for career opportunities as well as the growing popularity of diets high in processed foods from restaurants and fast food. Falling short of the recommended minimum intake of fruits and vegetables was the most prevalent factor related to unhealthy diet. Further studies are warranted to assess knowledge and attitude on their intake which could contribute to establishing strategies to improve the trend of healthy diet consumption.

Although dietary fats and fatty acids are vital nutrients, the type of fat along with the amount consumed have contrasting effects on overall health as well as substantial implications for prevention and treatment of chronic disease, including type 2 diabetes, cancer, respiratory diseases, and multiple sclerosis [29]. Furthermore, research indicates that dietary fats have distinct implications [30]. The latest dietary recommendations suggest replacing saturated fats with unsaturated fats [31]. Saturated fats, such as butter, raises the cholesterol level, which consequently increases the risk of heart disease. As per the recommendation of the American Heart Association, substituting saturated fats with vegetable oils (which contain linoleic acid, a polyunsaturated fat) is presumed to contribute in reducing cholesterol levels, improving overall heart health. Higher levels of linoleic acid are found in certain vegetable oils, such as sunflower and corn, whereas others like canola and olive have lower levels [32]. The healthier oil choice to be used for cooking is still debatable. A couple of recent reports have also obfuscated the relationship between saturated fat and cardiovascular disease. A meta-analysis of 72 studies with over 103,052 people revealed that there was insufficient evidence that saturated fats increased the risk of heart disease, although replacing them with polyunsaturated fat might actually reduce this risk [33–35]. The finding was also corroborated by other major studies which concluded that substituting saturated fat with polyunsaturated fats such as vegetable oils or high-fibre carbohydrates is the best approach for heart disease risk reduction, though notably substituting saturated fat with highly processed carbohydrates could possibly be counterproductive [36–39]. Our study found that most Omanis use vegetable oil for cooking and it is significantly correlated with raised cholesterol and abnormal waist-to-hip ratio. Moreover, there is a 90% probability for any Omani to be in a class of population who are mostly users of vegetable oil. This could signify an area to provide targeted interventions to target this particular class to reduce the causative NCD burden [40].

To accurately capture the complex interactions among dietary risk factors and other potential contributors to non-communicable disease NCD development, SEM) was employed. This methodology provided a comprehensive view of the interrelations between sociodemographic, behavioral, and metabolic factors, thus offering a nuanced representation of the diverse influences on NCD pathogenesis. The analysis revealed that dietary risk factors exert significant roles in the development of NCDs, both directly and indirectly affecting key biophysical and biochemical indicators. Notably, fruit and vegetable consumption primarily exhibited indirect effects, except for a direct impact on cholesterol levels. Additionally, frequent consumption of meals outside the home directly influenced blood glucose and total cholesterol levels, while the type of cooking oil used was directly associated with variations in waist-to-hip ratio, total cholesterol, and blood pressure.

This study is pioneering in its use of both LCA and SEM to explore the heterogeneity and intricate interplay of dietary risk factors with other indicators in NCD development. However, a potential limitation of this research is the susceptibility to recall bias, as the dietary data were collected through self-reported food recall questionnaires, relying solely on participants' reported intake. Another limitation was that this study only evaluated 3 dietary habits: intake of vegetables & fruits, eating out and type of oil as a result of data derived from the nutrition component of the WHO STEPS survey. Further research needs to be done to evaluate other strong associations with detrimental values for biomarkers of disease, inflammation and presence of non-communicable diseases including but not limited to such as high intake of sugar/carbohydrates and insufficient intake of w3 fatty acids.

## Conclusions

The detrimental impacts of behavioural and dietary risk factors on NCDs, along with the metabolic and physiological mechanisms that mediate these effects, have been well documented through prospective cohort studies and randomized controlled trials. Nonetheless, there remains a critical need to delineate the disease burden attributable to dietary risk factors specifically within the Omani context, necessitating comprehensive national-level analysis based on data indicating prevalent risk factors among adult Omanis [41]. Our study highlighted that the dietary intake recommendations set by the WHO were not met for most assessed variables. Furthermore, the results underscore the importance of developing tailored health promotion strategies that incorporate innovative processes and techniques tailored to the Omani demographic.

## Supplementary Information

Supplementary Material 1.

Supplementary Material 2.

## Data Availability

The datasets generated and/or analysed during the current study are not publicly available due to data sharing policies of Oman and the Ministry of Health but are available from the corresponding author on reasonable request.

## Abbreviations

AIC: Akaike’s Information Criterion
BIC: Bayesian Information Criterion
CVD: Cardiovascular diseases
DALY: Disability-adjusted life years
FAO: Food and Agricultural Organization
FBDG: Food-based dietary guidelines
GBD: Global burden of diseases
HDL: High-density Lipoprotein
LCA: Latent Class Analysis
NCD: Non-communicable diseases
SEM: Structural equation modelling
STEPS: STEPwise approach to risk factor surveillance
WHO: World Health Organization
