# Supplementary material for: Dietary risk factors for non-communicable diseases among Omani adults by latent class analysis and structural equation modelling
Source: BMC Nutr. 2025 Apr 24;11:84. doi: 10.1186/s40795-024-00987-y (PMC12020193; doi:10.1186/s40795-024-00987-y)
Supplement: Supplementary file 1 — Supplementary Material 1. [file 40795_2024_987_MOESM1_ESM.pdf]

Participant Identification Number |_|_|_| |_|_|_| |_|_|_|

CORE: Diet

The next questions ask about the fruits and vegetables that you usually eat. I have a nutrition card here that shows you some examples of local fruits and vegetables. Each picture represents the size of a serving. As you answer these questions please think of a typical week in the last year.

Question	Response	Code
In a typical week, on how many days do you **eat fruit**? *(USE SHOWCARD)*	Number of days<br>Don't Know 77 |_|_| *If Zero days, go to D3*	D1
How many **servings** of fruit do you eat on **one** of those days? *(USE SHOWCARD)*	Number of servings<br>Don't Know 77 |_|_|	D2
In a typical week, on how many days do you **eat vegetables**?*(USE SHOWCARD)*	Number of days<br>Don't Know 77 |_|_| *If Zero days, go to D5*	D3
How many **servings** of vegetables do you eat on one of those days? *(USE SHOWCARD)*	Number of servings<br>Don't know 77 |_|_|	D4

Dietary salt

With the next questions, we would like to learn more about salt in your diet. Dietary salt includes ordinary table salt, unrefined salt such as sea salt, iodized salt, salty stock cubes and powders, and salty sauces such as soya sauce or fish sauce (see show card). The following questions are on adding salt to the food right before you eat it, on how food is prepared in your home, on eating processed foods that are high in salt such as *[insert country specific examples]*, and questions on controlling your salt intake. Please answer the questions even if you consider yourself to eat a diet low in salt.

Question	Response		Code
How often do you **add salt or a salty sauce such as soya sauce** to your food right before you eat it or as you are eating it?<br>*(SELECT ONLY ONE)*<br>*(USE SHOWCARD)*	Always<br>Often<br>Sometimes<br>Rarely<br>Never<br>Don't know	1<br>2<br>3<br>4<br>5<br>77	D5
How often is **salt, salty seasoning or a salty sauce added** in cooking or preparing foods in your household?	Always<br>Often<br>Sometimes<br>Rarely<br>Never<br>Don't know	1<br>2<br>3<br>4<br>5<br>77	D6
How often do you eat **processed food high in salt**? By processed food high in salt, I mean foods that have been altered from their natural state, such as packaged salty snacks, canned salty food including pickles and preserves, salty food prepared at a fast food restaurant, cheese, bacon and processed meat *[add country specific examples].*<br>*[INSERT EXAMPLES]*<br>*(USE SHOWCARD)*	Always<br>Often<br>Sometimes<br>Rarely<br>Never<br>Don't know	1<br>2<br>3<br>4<br>5<br>77	D7
**How much salt or salty sauce** do you think you consume?	Far too much<br>Too much<br>Just the right amount<br>Too little<br>Far too little<br>Don't know	1<br>2<br>3<br>4<br>5<br>77	D8

**EXPANDED: Diet**		
**Question**	**Response**	**Code**
How important to you is **lowering the salt** in your diet?	Very important 1<br>Somewhat important 2<br>Not at all important 3<br>Don't know 77	D9
Do you think that too much salt or salty sauce in your diet could cause a **health problem**?	Yes 1<br>No 2<br>Don't know 77	D10
Do you do any of the following on a regular basis to **control your salt intake**?<br>*(RECORD FOR EACH)*		
Limit consumption of processed foods	Yes 1<br>No 2	D11a
Look at the salt or sodium content on food labels	Yes 1<br>No 2	D11b
Buy low salt/sodium alternatives	Yes 1<br>No 2	D11c
Use spices other than salt when cooking	Yes 1<br>No 2	D11d
Avoid eating foods prepared outside of a home	Yes 1<br>No 2	D11e
Do other things specifically to control your salt intake	Yes 1 *If Yes, go to D11other*<br>No 2	D11f
Other (please specify)	└─┴─┴─┴─┴─┴─┴─┘	D11other
The next questions ask about the oil or fat that is most often used for meal preparation in your household, and about meals that you eat outside a home.		
What type of **oil or fat is most often** used for meal preparation in your household?<br><br>*(USE SHOWCARD)*<br>*(SELECT ONLY ONE)*	Vegetable oil 1<br>Lard or suet 2<br>Butter or ghee 3<br>Margarine 4<br>Other 5 *If Other, go to D12 other*<br>None in particular 6<br>None used 7<br>Don't know 77	D12
	Other └─┴─┴─┴─┴─┴─┴─┘	D12other
On average, how many meals per week do you eat that were not prepared at a home? By meal, I mean breakfast, lunch and dinner.	Number<br>Don't know 77 └─┴─┘	D13
