# Supplementary material for: Dietary risk factors for non-communicable diseases among Omani adults by latent class analysis and structural equation modelling
Source: BMC Nutr. 2025 Apr 24;11:84. doi: 10.1186/s40795-024-00987-y (PMC12020193; doi:10.1186/s40795-024-00987-y)
Supplement: Supplementary file 2 — Supplementary Material 2. [file 40795_2024_987_MOESM2_ESM.pdf]

Table S1: Model selection, Akaike's information criterion and Bayesian information criterion

Model	N	df	AIC	BIC
**One class**	4,320	6	23290.87	23329.1
**Two class**	4,320	13	23224.59	23307.42

Table S2: Latent class marginal probabilities of dietary habits as indicators for waist-to-hip ratio (x10^{-2})

Class	Marginal probability		[95% Conf. Interval]
**1**	13.95	4.87	6.82 – 26.44
**2**	86.05	4.87	73.56 – 93.18

Table S3: Latent Class marginal means of dietary habits as indicators for waist-to-hip ratio (x10^{-2})

	Marginal mean		[95% Conf. Interval]
**Class 1**			
**Waist-to-hip ratio**			
**Normal**	21.67	8.17	9.72 – 41.53
**Abnormal**	78.33	8.17	58.47 – 90.28
**Class 2**			
**Waist-to-hip ratio**			
**Normal**	35.85	2.06	31.93 – 39.98
**Abnormal**	64.15	2.06	60.02 – 68.07

Table S4: Latent class marginal probabilities of dietary habits extended to sociodemographic factors ($x10^{-2}$)

Class	Margin	[95% Conf. Interval]
**1**	67.30	65.51 – 69.03
**2**	32.70	30.97 – 34.49

Table S5: Latent class marginal means of dietary habits extended to sociodemographic factors ($x10^{-2}$)

	Margin	95% CI	Margin	95% CI
**Class**	**Class 1**		**Class 2**	
**Eating Outside the home**				
0	39.96	38.10 - 41.85	78.75	76.32 - 80.99
1-3 times	45.28	43.42 - 47.16	18.91	16.78 - 21.25
4 or more times	14.76	13.49 - 16.12	2.34	1.62 - 3.37
**Fruit/Vegetable Intake**				
>5 servings of fruit/veg on average/day	39.75	37.95 - 41.59	32.44	29.93 - 35.05
<5 servings of fruit/veg on average/day	60.25	58.41 - 62.05	67.56	64.95 - 70.07
**Fat Used for Cooking**				
Vegetable oil	87.84	86.58 - 89.00	87.73	85.83 - 89.40
Butter	3.08	2.50 - 3.80	4.81	3.78 - 6.12
Other	9.07	8.07 - 10.19	7.46	6.16 - 9.01
**Sedentary Lifestyle**				
1	61.82	59.99 - 63.61	58.89	56.17 - 61.55
2	38.18	36.39 - 40.01	41.11	38.45 - 43.83
**Marital Status**				
Not Married	24.18	22.63 - 25.80	2.76	1.89 - 4.02
Married	73.62	71.95 - 75.22	72.25	69.75 - 74.61
Separated / Divorced	1.88	1.43 - 2.46	4.20	3.25 - 5.42
Widowed	0.32	0.15 - 0.68	20.79	18.66 - 23.10
**Age groups**				
18-29	36.19	34.41 - 38.01	3.52	2.49 - 4.95
30-39	40.96	39.15 - 42.81	10.34	8.55 - 12.45
40-49	19.04	17.58 - 20.60	21.40	19.15 - 23.83
50-59	3.24	2.61 - 4.02	27.59	25.20 - 30.11
60+	0.55	0.31 - 0.98	37.15	34.43 - 39.97
**Education level**				
No formal education	1.89	1.10 - 3.22	86.47	83.69 - 88.83
Preparatory or less	11.15	9.85 - 12.59	11.55	9.47 - 14.03
Secondary completed	55.73	53.75 - 57.69	1.98	1.08 - 3.61
University+	31.23	29.50 - 33.01	0.00	0.00 - 100.00
**Work status**				
Working in public sector	34.65	32.89 - 36.46	2.47	1.63 - 3.73
Working in private sector	12.40	11.22 - 13.69	7.41	6.02 - 9.09
Not working	52.94	51.07 - 54.81	90.12	88.11 - 91.82

Table S6: Latent class marginal probabilities of sociodemographic factors with blood glucose (x10^{-2})

Class	Margin	[95% Conf. Interval]
1	63.10	48.85 - 75.38
2	36.90	24.62 - 51.15

Table S7: Latent class marginal means of sociodemographic factors with blood glucose (x10^{-2})

	Margin	95% CI	Margin	95% CI
Class	Class 1		Class 2	
**Blood Glucose**				
1) Blood glucose <6.1	72.06	66.39 - 77.10	62.29	53.57 - 70.28
2) Blood glucose ≥ 6.1 and <7.0	21.26	17.58 - 25.46	5.32	1.46 - 17.62
3) Blood glucose ≥ 7.0	6.68	3.43 - 12.62	32.39	25.61 - 40.00

Table S8: Goodness of Fit

Goodness of fit statistics	Value
Root mean squared error of approximation	0.024
PCLOSE	1
Comparative fit index	0.974
Tucker Lewis index	0.947
Standardized root mean squared residual	0.015
Coefficient of determination	0.297
